# A universal strategy for regulating mRNA translation in prokaryotic and eukaryotic cells

**DOI:** 10.1093/nar/gkv290

**Published:** 2015-04-06

**Authors:** Jicong Cao, Manish Arha, Chaitanya Sudrik, Abhirup Mukherjee, Xia Wu, Ravi S. Kane

**Affiliations:** Department of Chemical and Biological Engineering, Center for Biotechnology and Interdisciplinary Studies, Rensselaer Polytechnic Institute, Troy, NY 12180, USA

## Abstract

We describe a simple strategy to control mRNA translation in both prokaryotic and eukaryotic cells which relies on a unique protein–RNA interaction. Specifically, we used the Pumilio/FBF (PUF) protein to repress translation by binding in between the ribosome binding site (RBS) and the start codon (in *Escherichia coli*), or by binding to the 5′ untranslated region of target mRNAs (in mammalian cells). The design principle is straightforward, the extent of translational repression can be tuned and the regulator is genetically encoded, enabling the construction of artificial signal cascades. We demonstrate that this approach can also be used to regulate polycistronic mRNAs; such regulation has rarely been achieved in previous reports. Since the regulator used in this study is a modular RNA-binding protein, which can be engineered to target different 8-nucleotide RNA sequences, our strategy could be used in the future to target endogenous mRNAs for regulating metabolic flows and signaling pathways in both prokaryotic and eukaryotic cells.

## INTRODUCTION

Advances in synthetic biology, especially in gene regulation, provide powerful tools to study biological systems as well as to develop complex artificial genetic processes (1–8). Gene expression can be regulated on both the transcriptional and translational levels. Most synthetic biology tools and systems focus on transcriptional regulation using DNA–protein interactions (4,9,10). However, in recent years, the advantages of translational regulation over transcriptional regulation and developments in RNA technology and nucleic acid engineering have accelerated the design and construction of RNA-based gene regulatory systems (6,11–17).

Compared with transcriptional regulation, translational regulation can result in more rapid changes in gene expression in response to stimuli. Moreover, in eukaryotic cells, such a regulatory approach may allow the synthesis of proteins at specific locations in cells. Despite these advantages, there are numerous challenges associated with engineering RNA-based regulators. For instance, since single-strand RNA can fold into complex secondary structures, the rate of translation of a target mRNA may be significantly impacted by the secondary structure of the 5′ untranslated region (5′-UTR) (18,19). Screening-based approaches to designing RNA switches can be time-consuming and laborious, and approaches using structure prediction software and other prediction tools may still require optimization (6,20–24). Moreover, the mechanisms of translation initiation are different in prokaryotic and eukaryotic cells, making it difficult to develop universal approaches for translational regulation.

Riboswitches and ribozymes have been widely used to regulate RNA translation and degradation in response to input ligands (12,22,25,26). Artificial riboswitches have been designed to regulate translation in both prokaryotic and eukaryotic cells, but the design strategies and the locations on target RNAs are different (12). Recently, there has been an increasing interest in building translational regulators using proteins as the input ligands—protein–RNA complex-based translational regulators (25,27–29). The advantage of protein-based regulatory systems is that the output (protein) can serve as the input ligand for other translational switches, thereby facilitating the construction of complex genetic circuits or signal cascades.

We believe that an ideal translational regulation system should have the following characteristics: first, the regulators should be capable of binding RNA elements regardless of their secondary structure. Second, one should be able to synthesize or express the input ligands within cells to enable the construction of artificial signal cascades. Third, the regulation strategy should work in both prokaryotic and eukaryotic cells. Fourth, one should be able to apply the strategy to a large number of regulator/reporter pairs. Fifth, one should be able to use the strategy to target endogenous RNAs.

**Figure 1.**
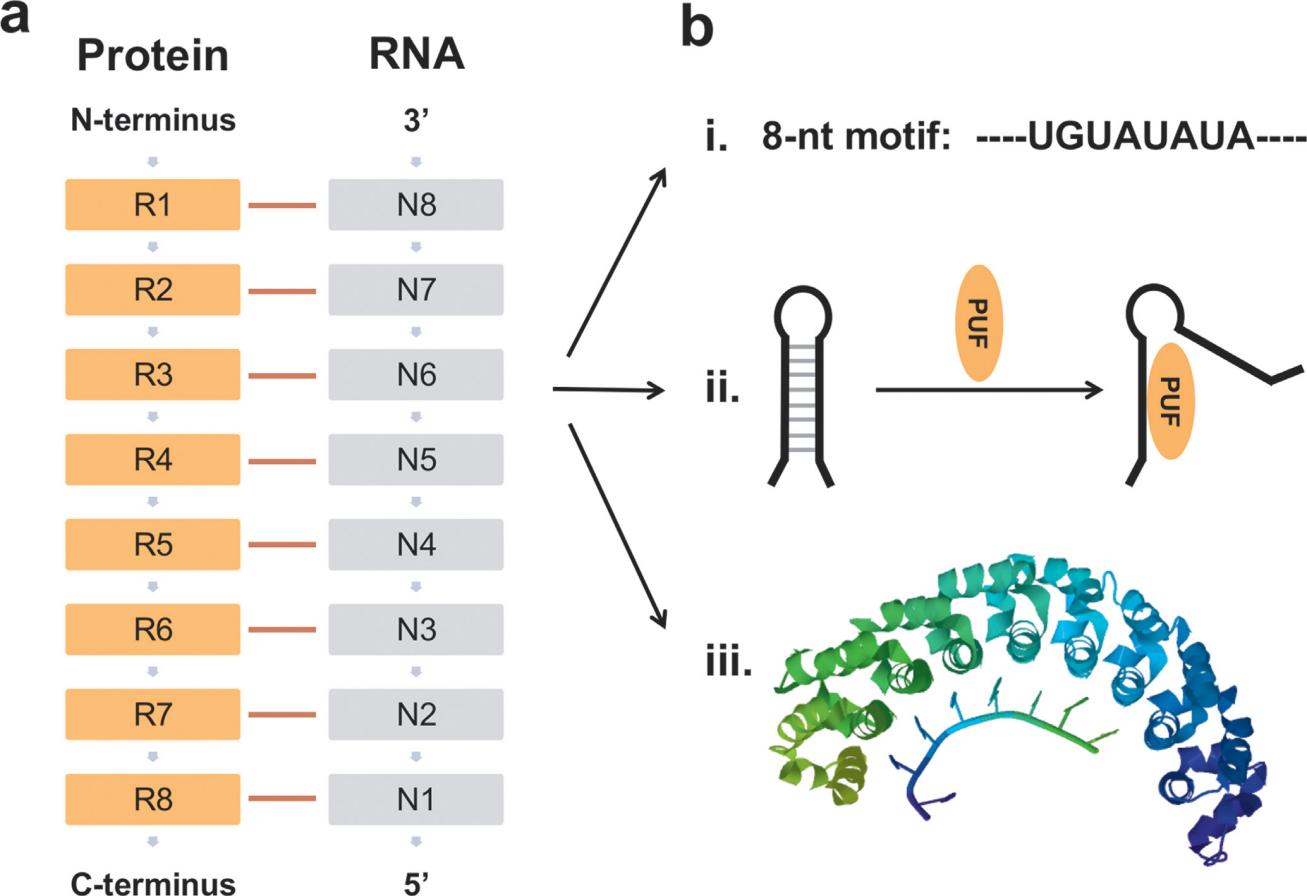
The interaction between the PUF domain and its target RNA. (**a**) The eight RNA-recognition repeats of the PUF domain (R1-R8) recognize eight consecutive RNA nucleotides (N8–N1), with each repeat binding to 1 nucleotide. (**b**) The unique architecture of the PUF–NRE interaction. (i) NRE contains only 8 nucleotides. (ii) PUF can bind to target RNA having complex structures in the unbound state. (iii) Cartoon showing PUF (PDB ID: 1m8W) (29) bound to a target RNA. The target RNA is typically unfolded and does not have a hairpin structure in the bound state.

We reasoned that Pumilio/FBF (PUF) domains would be promising candidates for such a system (30–32) (Figure 1a). The RNA-binding domain of PUF proteins contains eight modular RNA-binding repeats, each composed of 36 amino acids; each repeat binds one RNA base independently (30,31,33). The well-studied and most commonly used PUF domain is the human Pumilio1 homology domain, which recognizes the sequence UGUAUAUA, known as the Nanos response element (NRE). Biochemical studies and crystal structure data reveal that two amino acids in each repeat are primarily responsible for the RNA base recognition, and the specificity of recognition can be changed to any of the four RNA bases by introducing proper site mutations (31,33–35). In principle, artificial PUF domains can be engineered to target any 8-nucleotide (8-nt) RNA sequence. For example, Cheong and Hall have engineered PUF mutants that bind to different 8-nt RNA sequences with high specificity (34).

In previous studies, PUF domains have been used as tethers to target endogenous RNAs and to regulate a variety of post-transcriptional processes when fused with functional modules (32,35–37). For example, when PUF was fused with an RNA endonuclease domain PIN, it could digest endogenous RNAs in *Escherichia coli* (38). Another example is PUF-based RNA splicing, when PUF was fused with RNA splicing regulatory elements (36). Our group had previously used PUF domain to control both mRNA translation activation and repression in mammalian cells (39). As we noted, in most of the previous studies, PUF domains were used as tethers to regulate post-transcriptional pro-cesses of target RNAs. However, in this study, we explored the potentials of PUF domains to control RNA translation based on its unique interaction architecture with its target RNA, where PUF domains alone act as translational repressors when binding to the 5′-UTR of target prokaryotic and eukaryotic mRNAs. Compared with other protein or peptide ligands, PUF domains have several advantages (Figure 1b). First, unlike most RNA aptamers, which usually contain 30–40 nucleotides, the RNA target (NRE) of PUF domains contains only 8 nt (34). Second, PUF domains can recognize target RNAs even when every base of the target RNA is paired with a complementary base in the unbound state (31). Third, PUF domains do not require the target RNA to be folded into a specific secondary structure; the RNA target is exclusively single-stranded when interacting with the PUF domain.

In this work, we have demonstrated a universal approach to control mRNA translation using PUF domains in both prokaryotic and eukaryotic cells. In prokaryotic cells, we demonstrated the ability to repress translation by the binding of PUF in between the ribosomal binding site (RBS) and the start codon (AUG) of the gene of interest without requiring any special 5′-UTR design. Moreover, we demonstrated the use of this approach to regulate the translation of individual cistron of a bicistronic mRNA, which has rarely been achieved in previous reports. In eukaryotic cells, PUF domain significantly repressed target gene expression upon binding to the 5′-UTR of the target mRNA. Since PUF domains are modular and designable, the approaches we developed in this study could be potentially used to control endogenous mRNA translation in prokaryotic and eukaryotic cells in the future.

## MATERIALS AND METHODS

### DNA constructions

All the primers and DNA oligonucleotides used in this study were purchased from Integrated DNA Technologies, Inc. (IL, USA). Nucleotide sequences of the primers and the oligonucleotides are listed in Supplementary Tables S1–S3. We used *E. coli* expression vectors pET Duet-1 and pRSF Duet-1 (EMD Millipore, MA, USA) to make the required DNA constructs for carrying out experiments in prokaryotic system. Briefly, to construct pRSF-PUF the PUF(wt) was amplified by polymerase chain reaction (PCR) from pTYB3-PUM1-HD which was kind gift by Dr Traci Hall (Addgene plasmid # 17543) and cloned between NcoI and BamHI sites of plasmid pRSF Duet-1. The GFP gene required to make the reporter constructs, CON-GFP and NRE (7 or 8 or 9 or 10 or 11 or 12)-GFP, was amplified by PCR from plasmid pEGFP-C1 (Clontech, CA, USA) and cloned into XbaI and BamHI sites of plasmid pET Duet-1. We also constructed a prokaryotic bicistronic reporter plasmid that encodes for GFP as an upstream cistron and mCherry as a downstream cistron. The CON or NRE(8)- mCherry encoding sequence required for the bicistronic constructs was PCR amplified from plasmid pmCherry-N1 (Clontech, CA, USA) and cloned into BamHI and XhoI sites downstream of either CON-GFP or NRE(8)-GFP. We used mammalian expression vector pVITRO2-hygro-mcs (Invivogen Inc., CA, USA) to make the DNA constructs and conduct experiments in eukaryotic system. Briefly, to make a GFP-based reporter we inserted oligonucleotides coding for NRE 1X Position 1, NRE 3X Position 1, NRE 1X Position 9 and NRE 6-2/7-2 1X Position 1 in the 5′-UTR of plasmid pCII-1(28) in between sites EcoRI and BamHI. Similarly, we also made luciferase-based reporters where the nucleotide sequence encoding GFP in the reporter plasmids described above was replaced by a nucleotide sequence encoding luciferase. The luciferase gene was obtained by PCR amplification from plasmid pGL3 Promoter (Promega Corporation, WI, USA) and cloned in between BglII and XhoI sites. For construction of plasmids pPUF(wt) and pPUF(6-2/7-2), the PUF(wt) and PUF(6-2/7-2) coding sequences were amplified from pTYB3-PUM1-HD and pGly-PUF(6-2/7-2) (kindly provided by Dr Zefeng Wang) and cloned into BglII and BstBI sites of pVITRO2-hygro-mcs. Construction of plasmid pλ-LacZ and plasmid pMS2 has been described previously (40).

### Translational repression assay in bacteria

Rosetta 2(DE3) cells (Millipore Corporation) were grown in Lysogeny broth (LB) medium. Cells co-transformed with the reporter and regulator plasmids were grown overnight in LB medium at 37°C, 225 rpm. Next morning, 2 ml of fresh LB medium was inoculated with 50-μl overnight culture in a 24-well deep well plate covered by a gas permeable film and grown at 37°C, 225 rpm for 6 h (Optical density of the culture at 600 nm (OD_600_) was about 0.5). Isopropyl β-D-1-thiogalactopyranoside (IPTG) was added into each well to a final concentration of 0.5 mM and the cells were grown for additional 2.5 h. Optical density was read at 600 nm. For monocistronic experiments, GFP fluorescence was measured using VICTOR X3 Multilabel Plate Reader (PerkinElmer) and the excitation and emission wavelengths for GFP were set at 485/528 nm. For bicistronic experiments, GFP and mCherry fluorescence was measured using SpectraMax 5 (Molecular Devices), and the excitation and emission wavelengths for GFP were set at 485/538 nm and the excitation and emission wavelengths for mCherry were set at 584/612 nm. All experiments were performed in triplicates.

### Translation repression assay in mammalian cells

Human embryonic kidney (HEK) 293T cells (ATCC, VA, USA) were grown in polystyrene flasks in Dulbecco's Modified Eagle's Medium (Life Technologies Corporation, CA, USA) supplemented with 10% heat-inactivated fetal bovine serum and 1% penicillin/streptomycin (Life Technologies Corporation, CA, USA) at 37°C and 5% CO2. When the cells were 80–90% confluent, cells were harvested with 0.05% trypsin (Life Technologies Corporation, CA, USA) for transfection. For microscope experiments, HEK 293T cells were plated into 96-well plates at a density of 60 000 cells per well the day before transfection. For each well, 100-ng reporter plasmids and 50-ng regulator plasmids were mixed with 0.5-μl Lipofectamine 2000 reagent (Life Technologies Corporation, CA, USA) and used for transfection. After 48 h, the cells were observed under a fluorescent microscope with a 4X objective with either the green or red filter. For luciferase assay, HEK 293T cells were plated into 96-well plates at a density of 60 000 cells per well the day before transfection. For each well, 100-ng regulator plasmids were mixed with 0.5-μl Lipofectamine 2000 reagent and used for transfection. After 48 h, the luciferase activity of the transfected cells was determined using Luciferase Assay Kit or Dual-Luciferase Reporter Assay System (Promega Corporation, WI, USA) following the manufacturer's instructions. For western blotting assay, the HEK 293T cells co-transfected with reporter and regulator plasmids were lysed and resolved using sodium dodecyl sulphate-polyacrylamide gel electrophoresis. The lysate was then transferred from the gel onto a polyvinylidene fluoride (PVDF) membrane and probed using anti-HA (sc-7392) primary antibody (Santa Cruz Biotechnology, Inc., TX, USA) or anti-Glyceraldehyde 3-phosphate dehydrogenase (ab8245) primary antibody (Abcam) and anti-IgG (115-035-146) secondary antibody (Jackson Immunoresearch Laboratories). The intensity of the bands was analyzed using ImageJ.

### RNA extraction and quantitative real-time PCR

The total cellular RNA was extracted from various cell samples by using RNeasy Mini Kit (Qiagen Inc., CA, USA) as per the manufacturer's guidelines. Nanodrop ND-1000 was used to quantify the RNA and 1 μg of the RNA from each sample was used for the synthesis of cDNA by using ImProm-II Reverse Transcription system (Promega Corporation, WI, USA) as per the manufacturer's instructions. The cDNA was diluted 5-fold and stored at −20°C. Primers for luciferase, EGFP, rrsA (prokaryotic housekeeping gene) and 18S (eukaryotic housekeeping gene) were purchased from Integrated DNA Technologies Inc. (IL, USA). TaqMan probes for luciferase, EGFP, rrsA and 18S were synthesized by BioSearch Technologies (CA, USA). A list of the primers and probes and their nucleotide sequence is given in the Supplementary data. To set up quantitative real-time (QRT)-PCR reactions, Brilliant QRT-PCR Master Mix (Agilent Technologies Inc., CA, USA) was used as per the manufacturer's guidelines. Briefly, 0.2 μM of the primers and TaqMan probe, 1 μl of diluted cDNA as template and 12.5 μl of master mix were used in a total reaction volume of 25 μl. All the samples and no-template controls were run in triplicate in a 96-well plate on LightCycler 489 (Roche, Basel, Switzerland) by using a two-step cycling protocol where initial incubation is at 95°C for 10 min, followed by 70 amplification cycles (95°C for 15 s, 60°C for 1 min). The QRT-PCR data analysis presented in this study is based on three independent experiments.

## RESULTS

### Repression of prokaryotic mRNA translation relying on PUF–NRE interaction in *E. coli*

In prokaryotic cells, mRNA contains three primary elements: the 5′-UTR, the coding sequence starting with AUG and the 3′-UTR. The RBS contains the six-base consensus sequence AGGAGG in *E. coli* (22,41,42). Translation is initiated through an interaction between the 16S ribosomal RNA (rRNA) and the RBS that lies in the 5′-UTR of the mRNA. Previous reports showed that masking the RBS using complementary RNA fragments could repress translation by preventing 16S rRNA/ribosome binding (12,22,23,43). However, such a small molecule or protein inducible system is difficult for rational design and usually laborious screening processes are needed to construct a riboswitch or riboregulator with a desired secondary structure. Belmont and Niles screened an aptamer against the TetR protein and found that binding TetR protein to the aptamer located upstream of the RBS resulted in translational repression (2-fold reduction) (29). The fact that the RBS is generally located 8 bases upstream of the start codon for efficient translation in prokaryotic cells limits the ability to insert aptamers immediately downstream of the RBS (41,42,44). However, since the RNA target of PUF contains only 8 nt, we hypothesized that the binding of PUF in between the RBS and the AUG start codon might repress translation by sterically hindering ribosome access to the RBS.

**Figure 2.**
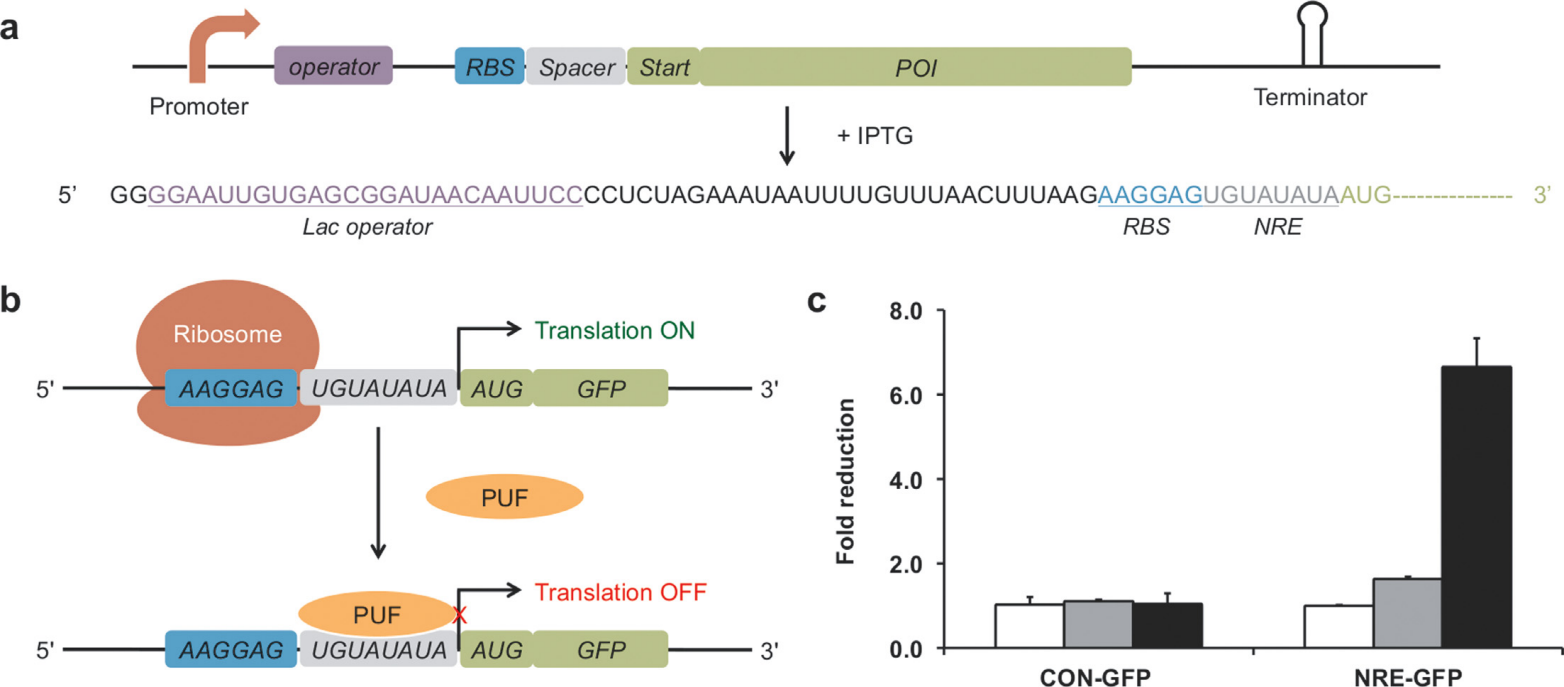
Repression of prokaryotic mRNA translation relying on the PUF–NRE interaction in *E. coli*. (**a**) The design of the reporter and the RNA sequence of the 5′-UTR of the reporter. POI: protein of interest (e.g. GFP). (**b**) A schematic illustrating the mechanism of translational repression. PUF binds to the PUF binding site in between the RBS and AUG of the reporter, thereby resulting in the repression of translation. (**c**) The influence of the PUF on the expression of GFP. The reporter plasmid NRE-GFP contains one PUF binding site, whereas the control reporter plasmid CON-GFP does not. The experiments were performed without regulator (white bars), with LacZ (gray bars) and with PUF (black bars). Fold reductions were calculated as the ratio of normalized GFP fluorescence for the reporter alone to that in the presence of PUF or LacZ. Values are the means of three independent experiments. Error bars show standard deviation.

*Escherichia coli* was selected as the model prokaryotic organism to test our hypothesis. We constructed a GFP reporter NRE-GFP, which contained an 11-amino-acid ssrA tag, AANDENYALVA, at the C-terminus of GFP to decrease the half-life of the fusion protein (Figure 2a). One NRE was inserted in between the RBS and translation initiation codon (AUG). The reporter construct was cloned in the pET vector with a copy number of ∼40, and the PUF domain was cloned in the pRSF vector with a copy number of ∼100 in *E. coli*. The two plasmids were co-transformed into Rosetta BL21 (DE3) cells, which contain tRNAs for rare codons in *E. coli*. GFP fluorescence was normalized with cell density (OD600). As predicted, we observed a 6-fold reduction in GFP fluorescence for the cells in the presence of PUF relative to that in the absence of PUF (Figure 2b and c). We also observed that the expression of a control protein, β-galactosidase (LacZ), did not result in repression of the translation of NRE-GFP. Since only 8 nt are enough for PUF binding, it was important to test the specificity of the system. We constructed a control GFP reporter CON-GFP in which the NRE sequence was replaced by an 8-nt sequence GAUAUACC (CON) modified from the pET vector. No significant change in GFP fluorescence was observed for cells co-transformed with plasmids encoding PUF and this control reporter.

**Figure 3.**
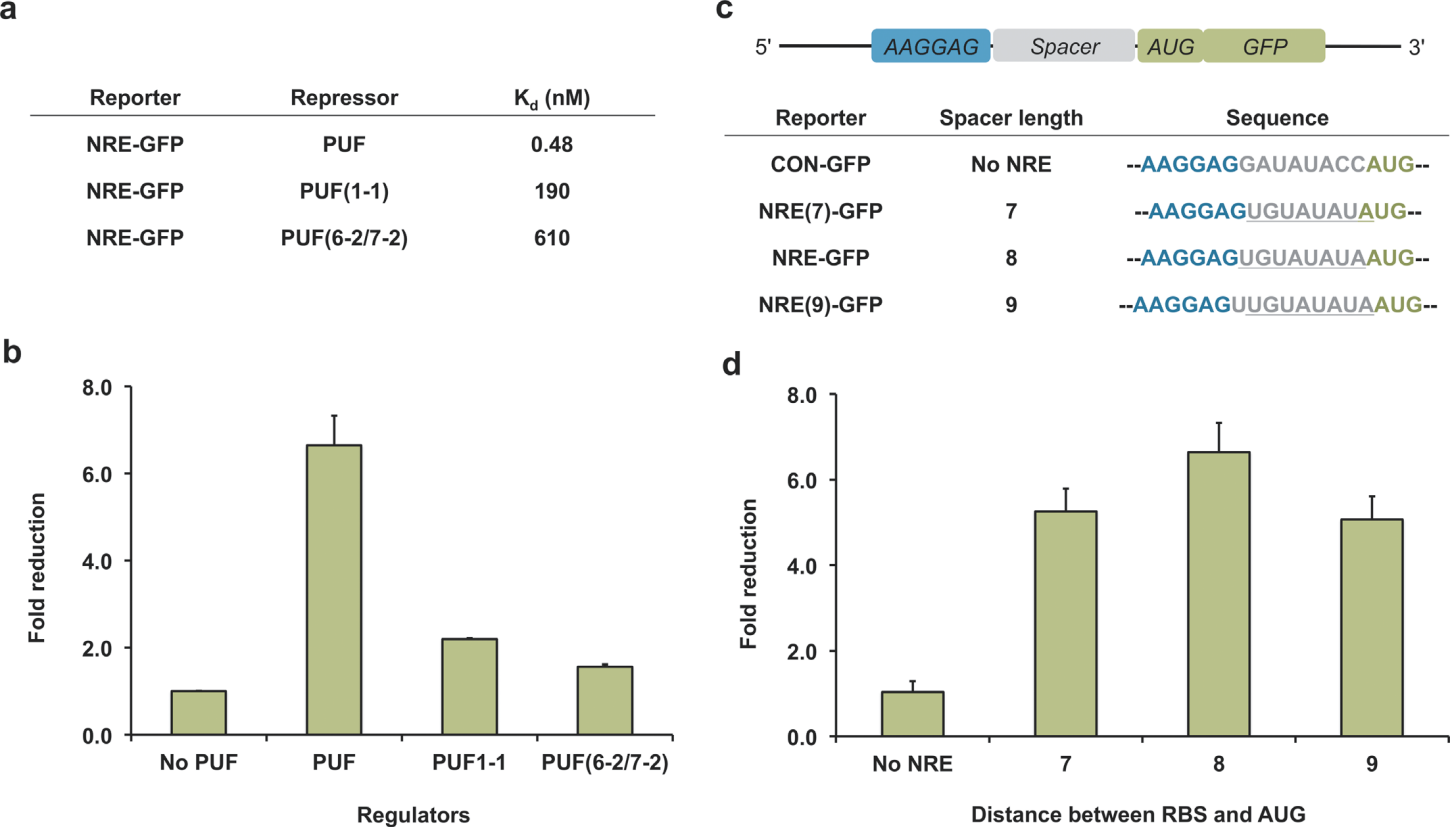
The influence of the *K*_d_ of the PUF–NRE complex and the length of the spacer on the extent of translational repression. (**a**) The affinities of wild type and mutant PUF domains for the NRE sequence. The data are from (34). (**b**) The influence of the value of *K*_d_ on the extent of translational repression. The reporter was NRE-GFP, which contained the NRE sequence. (**c**) Schematic representation of the reporters with spacers of different lengths and the sequence of the spacers. The sequence of NRE is underlined. (**d**) The influence of spacer length on the PUF-based translational repression of GFP. The distance between RBS and AUG of the reporter plasmids NRE(7)-GFP, NRE-GFP and NRE(9)-GFP is 7, 8 and 9, respectively. Values are the means of three independent experiments. Error bars show standard deviation.

We next tested the ability to tune the extent of translational repression by using PUF mutants, which differ in their affinity for the NRE sequence. The dissociation constant (*K*_d_) for the binding of PUF, PUF(1-1) and PUF(6-2/7-2) to NRE is 0.48, 190 and 610 nM, respectively (Figure 3a) (34). We found that the extent of translational repression was lower for PUF mutants with higher values of *K*_d_ (Figure 3b). These results indicate that we can use PUF mutants to tune the extent of translational repression.

We also tested the influence of the length of the spacer between the RBS and AUG on PUF-mediated repression. Since the spacer length often varies between 7 and 9 in endogenous genes (41,42), we constructed additional GFP reporters containing 7 (NRE(7)-GFP) and 9 (NRE(9)-GFP) nucleotides in between the RBS and AUG (Figure 3c). To construct NRE(7)-GFP, we used the fact that the last nucleotide in the NRE sequence (A) was identical to the first nucleotide in the AUG start codon. We observed a 5-fold translational repression for both of these reporters, similar to that for the NRE-GFP reporter (Figure 3d). Previous studies suggested that gene expression decreases significantly when the distance between the RBS and AUG is too long (41,42,45). Indeed, we constructed reporters containing longer spacers (10, 11, 12 nucleotides) and found GFP expression to be significantly lower; nonetheless, we still observed repression of GFP expression for these reporters (Supplementary Figure S1). We concluded that binding PUF domains to the spacer is a general approach for repressing gene expression.

### Repression of translation of an individual cistron in a bicistronic reporter based on PUF–NRE interaction

One key characteristic of prokaryotic mRNAs is that they can be polycistronic. A polycistronic mRNA contains two or more cistrons, each of which can be translated to an individual protein independently. Consequently, more than one protein can be produced from the same polycistronic mRNA. While rapid developments in synthetic biology have provided scientists with numerous systems and tools to control gene expression including transcriptional activators/repressors, riboswitches and antisense RNAs, (11) control of individual gene expression in polycistronic mRNAs has rarely been attempted. Although 5′-UTR design has previously enabled control of the expression of the primary cistron (12,22,23), limited success has been achieved in designing a riboswitch at the intercistronic region. We reasoned that the PUF-based repression approach might be useful for such a system, as PUF–NRE interaction can result in the unwinding of complex RNA secondary structures (31).

**Figure 4.**
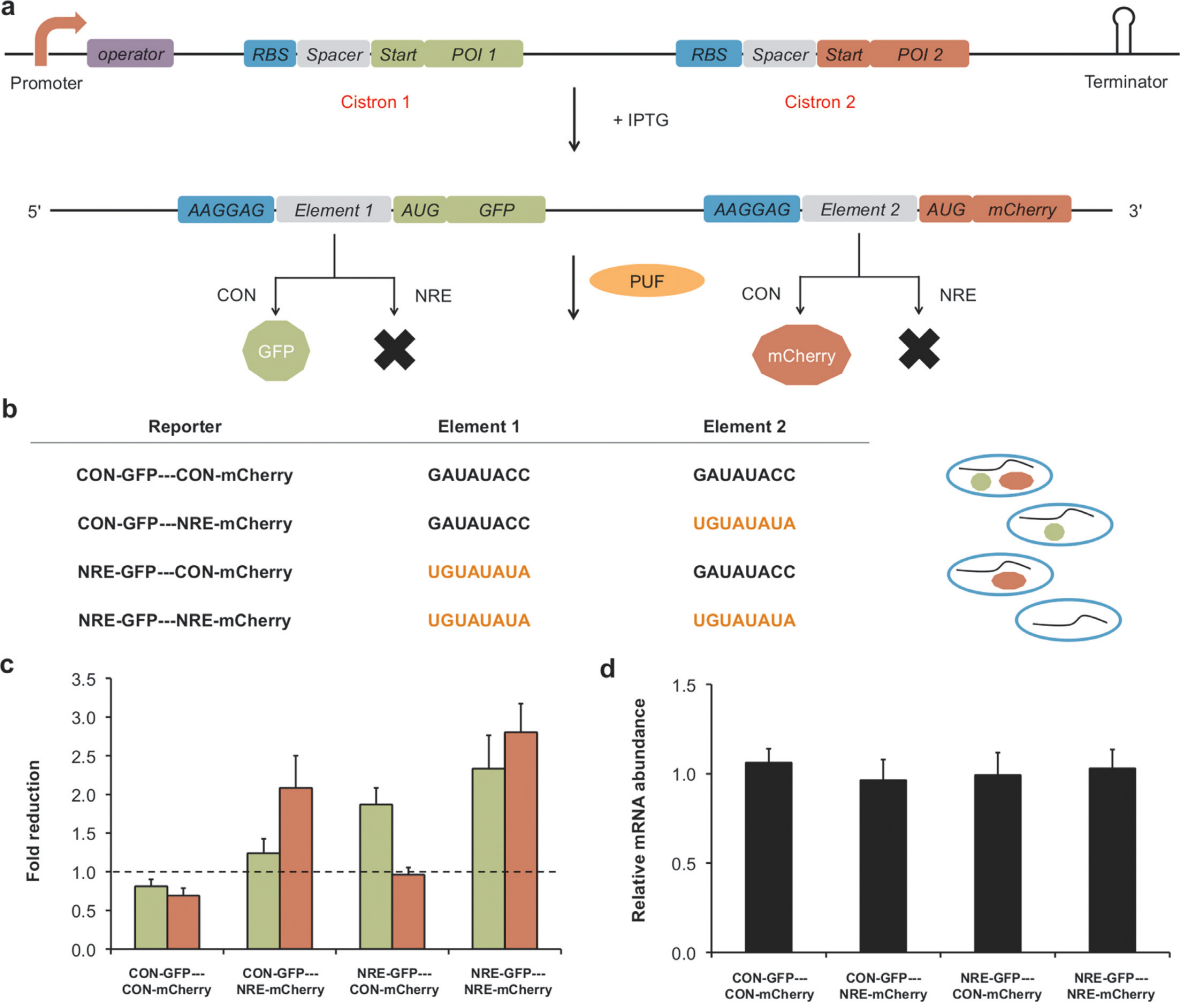
Repression of translation of an individual cistron in a bicistronic reporter relying on the PUF–NRE interaction. (**a**) A schematic illustrating the mechanism of translational repression of the bicistronic reporter. The bicistronic mRNA was transcribed from the reporter plasmids upon IPTG induction. The translation of the cistron (GFP or mCherry) is repressed if there is an NRE in between the RBS and AUG of the reporter, whereas the translation is not repressed if there is a CON in between the RBS and AUG. (**b**) Table indicating the RNA sequence of the 5′-UTR for the four bicistronic reporters. The cartoon on the right represents protein expression in the presence of PUF. (**c**) The influence of the PUF on the expression of GFP and mCherry. The fold reduction of normalized GFP fluorescence is in green bars and that of normalized mCherry fluorescence is in red bars. (**d**) The influence of PUF on the transcript level of the reporter mRNA analyzed using QRT-PCR. Relative mRNA abundance was calculated as the ratio of the normalized reporter transcript level in the absence of PUF to that in the presence of PUF. Values are the means of three independent experiments. Error bars show standard deviation.

To test the potential of the PUF-based repression system, we constructed a bicistronic reporter encoding two different fluorescence proteins, GFP (cistron 1) and mCherry (cistron 2) (Figure 4a). Each cistron contained its own RBS, spacer and the ORF (open reading frame) of one fluorescent protein. We inserted either CON or NRE as the spacer for each cistron to make four different bi-cistronic reporters (Figure 4b). We co-transformed *E. coli* with plasmids containing one of the reporters and plasmids containing either PUF or an empty vector. We observed that the expression of the cistron containing NRE decreased in the presence of PUF, whereas expression of the cistron containing CON did not for all the four reporters (Figure 4c). QRT-PCR results revealed that the amount of the reporter mRNA remained essentially unchanged, whereas there were predicted changes at the protein level (Figure 4d). These results confirmed that the repression was not due to a decrease in the level of reporter mRNA and are consistent with a PUF-based translational repression mechanism. We note that previous reports observed translational coupling effects, where the expression of the cistron 2 decreased when there was a significant repression of cistron 1. For example, Levin-Karp *et al*. observed 4- to 15-fold reduction of cistron 2 expression when the expression of cistron 1 was inhibited by 200-fold (46). They found that the coupling effects depended on the genetic context and were noticeable when the expression of cistron 1 was strongly inhibited. We did not observe significant translation coupling for modest repression of cistron 1. This approach provides a novel way to regulate the expression of individual genes of endogenous polycistronic mRNAs.

### Repression of eukaryotic mRNA translation based on PUF–NRE interaction in HEK 293T cells

The mRNA structure and translation initiation mechanism in eukaryotic cells are different from that in prokaryotic cells. Eukaryotic mRNAs contain five elements: 5′ 7-methylguanosine cap, 5′-UTR, coding region, 3′-UTR and poly(A) tail (19,47). The first step of translation initiation involves the circularization of the mRNA: eukaryotic initiation factor 4E (eIF4E) binds to the 5′ cap, the poly(A) binding protein (PABP) binds to the poly(A) tail at 3′ end and eIF4G interacts with eIF4E and PABP simultaneously to circularize the mRNA (47). The small ribosomal unit 40S binds to the circularized mRNA and forms the 43S pre-initiation complex together with other factors (47,48). The resulting complex scans the mRNA from the 5′ end until it finds the first AUG in an optimized context, known as the Kozak sequence (19,47). The 60S subunit joins with the 43S complex, the initiation factors are released and the resulting 80S ribosome initiates translation (47). As suggested by previous studies, binding small molecules or proteins to an aptamer located in the 5′-UTR of the target mRNA can result in the repression of translation by preventing ribosome binding or scanning (20,25,27,28,49). Although it is relatively easier to design a translational repression switch in eukaryotic cells than in prokaryotic cells, the design of an effective switch still requires considerable effort. In previous work, involving attempts to repress translation of a target mRNA using ligand–RNA interactions, we and others have shown that the thermodynamic stability of a ligand-binding aptamer and the location and the number of repeats of the aptamer on the 5′-UTR have a significant impact on the extent of translational repression (28,50). However the basal gene expression level was also correlated with the thermodynamic stability of the inserted aptamer/hairpin and the number of repeats of the aptamer, and only the cap-proximal location of the hairpin resulted in effective repression of target gene expression (28,51). These constraints limit the flexibility in reporter design.

**Figure 5.**
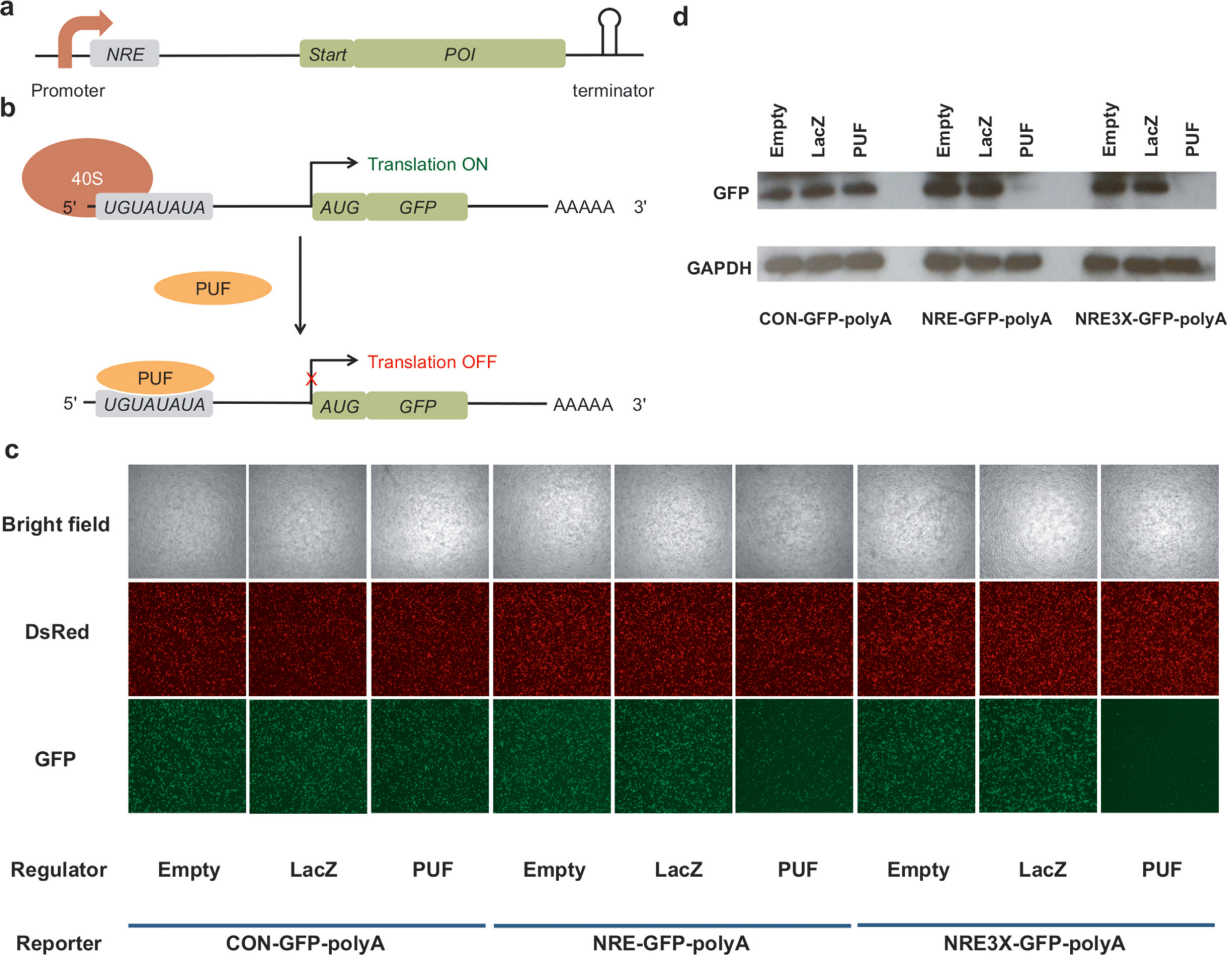
Repression of eukaryotic mRNA translation relying on the PUF–NRE interaction in HEK 293T cells. (**a**) The design of the GFP reporter. (**b**) A schematic illustrating the mechanism of translational repression. PUF binds to the PUF binding site located on 5′-UTR of the reporter, thereby resulting in the repression of translation. (**c**) Fluorescence micrographs of cells co-transfected with the plasmids coding for CON-GFP-polyA or NRE-GFP-polyA or NRE3X-GFP-polyA and those coding for regulators. (**d**) Repression of translation analyzed using immunoblotting. Cell lysate containing10 μg protein was loaded in each lane.

We chose HEK 293T cells as the model eukaryotic cells. We used a mammalian expression vector pVitro2 to construct the reporter, which contained two multiple cloning sites (MCSs). To construct the reporter plasmid, we inserted the sequence coding for GFP into MCS1 and that for CFP into MCS2. Similarly, we constructed the regulator plasmid by inserting the coding sequence for PUF into MCS1 and that for DsRed into MCS2. NRE or CON was inserted into the 5′-UTR of the GFP reporter mRNA, to construct the reporter NRE-GFP-polyA or CON-GFP-polyA, respectively (Figure 5a). We hypothesized that binding of PUF in the 5′-UTR of the reporter mRNA could repress translation by preventing ribosome binding (Figure 5b). We co-transfected the reporter plasmid and the regulator plasmid into HEK 293T cells, and characterized the cells by fluorescence microscopy 48 h post transfection. We found greater gene expression repression in the presence of PUF than that in the presence of the control protein ligand LacZ for the reporter NRE-GFP-polyA. In contrast, no repression was observed in the presence of PUF for the reporter CON-GFP-polyA (Figure 5c). We also constructed the reporter NRE3X-GFP-polyA, which consisted of three tandem NREs in the 5′-UTR. Even greater repression was observed for NRE3X-GFP-polyA and this result was consistent with previous studies where inserting additional aptamers/binding sites enhanced repression (28). Analysis by western blotting (Figure 5d) confirmed that the decrease in fluorescence was a result of decrease in the amount of GFP protein. Consistent with the fluorescent images, we observed a 4-fold reduction in band intensity for the reporter with one NRE and 13-fold reduction for the reporter with three NREs (Supplementary Table S4). We also quantified the reporter mRNA levels using QRT-PCR and found there to be no significant difference for the samples co-transfected with or without PUF (Supplementary Figure S2). These results confirmed that the repression of gene expression occurred at the translational level. We also observed that the MS2 coat protein, a commonly used RNA-binding protein, did not repress the translation of GFP, which further demonstrated the specificity of the system (Supplementary Figure S3 and Supplementary Table S5).

Compared with small molecule or other protein-based repression systems, our PUF-based repression system exhibited enhanced repression without the need to design the location of binding sites or the free energy of unfolding of inserted aptamers. Notably, previous studies suggest that while increasing the number of ligand binding repeats can enhance repression, they also result in a decrease in gene expression in the absence of ligand (28). However, in our system, increasing the number of the PUF binding sites does not significantly decrease gene expression. Similar results were observed when the GFP reporter was replaced by another reporter firefly luciferase (Luc), establishing the generality of the approach (Supplementary Figure S4).

## DISCUSSION

In this study, we have developed an approach to regulate mRNA translation in both prokaryotic and eukaryotic cells. The interaction between PUF and NRE enables regulation of translation upon binding to the 5′-UTR of target mRNAs. Unlike small molecule inducers, PUF domains are genetically encoded, and can be used as both input and output signals to pass along the genetic information. The PUF-based translational switches relying on protein–RNA interactions can be combined with widely used transcriptional switches relying on protein–DNA interactions to construct complex genetic circuits and signal cascades.

One key advantage of this approach is that we did not need to optimize or screen the 5′-UTR of the reporter, as in the common strategies for the design of translation regulators such as riboswitches or riboregulators. For example, one approach to design riboswitches that control translation in *E. coli* involves the elegant design or screening of a 5′-UTR that undergoes inducible change in the mRNA secondary structure to reveal or conceal the RBS to regulate translation (12,22). In contrast, our approach does not require any complicated design or optimization of the 5′-UTR. Indeed, we derived the sequence of the 5′-UTR of the reporter from a widely used commercial plasmid PET, with the only modification involving the insertion of the NRE in between the RBS and AUG (Supplementary Figure S5). We note that one advantage of riboswitches and riboregulators is that one can tune the strength of expression in a large range by rational design or high-throughput screening. In our work, engineering both PUFs and their target RNA binding systems can also enable tunable control of protein translation/repression by varying the affinity of the PUF for the target RNA. In mammalian cells, the 5′-UTR used in this report was from the commercially available vector pVitro2 and we only inserted one NRE near the 5′ cap. Moreover, unlike other protein or peptide ligands, which cannot effectively repress gene expression when the aptamer is not located near the cap, the PUF-based repressor can repress mRNA translation even when located 2,000 nucleotides away from the 5′ cap (39). One possible explanation for this result might be that the protein-RNA interaction of PUF-NRE is resistant to the RNA helicase associated with the 43S pre-initiation complexes.

In its current implementation, the binding of PUF to its target cannot be regulated using small molecules. One could readily design a system in which PUF expression is regulated using small molecules, thereby enabling inducible repression. In future work, it would be interesting to design allosteric fusion proteins in which the binding of PUF to its target site can be directly regulated using small molecules or other signals such as light (52,53). In addition, PUF domains are modular RNA-binding domains, which can be modified easily to target any 8-nt RNA sequence. Therefore, in the future, artificial PUF domains could be employed to control the translation of endogenous mRNAs and directly applicable to spatiotemporal gene knockdown for metabolic control and signaling pathway regulation.

## SUPPLEMENTARY DATA

Supplementary Data are available at NAR Online.

SUPPLEMENTARY DATA
