# Supplementary material for: A universal strategy for regulating mRNA translation in prokaryotic and eukaryotic cells
Source: Nucleic Acids Res. 2015 Apr 6;43(8):4353–62. doi: 10.1093/nar/gkv290 (PMC4417184; doi:10.1093/nar/gkv290)
Supplement: SUPPLEMENTARY DATA [file supp_43_8_4353__index.html]

A universal strategy for regulating mRNA translation in prokaryotic and eukaryotic cells — A universal strategy for regulating mRNA translation in prokaryotic and eukaryotic cells — SUPPLEMENTARY DATA 

# A universal strategy for regulating mRNA translation in prokaryotic and eukaryotic cells

## SUPPLEMENTARY DATA

**Files in this Data Supplement:**

- SUPPLEMENTARY DATA
