# Supplementary material for: A universal strategy for regulating mRNA translation in prokaryotic and eukaryotic cells
Source: Nucleic Acids Res. 2015 Apr 6;43(8):4353–62. doi: 10.1093/nar/gkv290 (PMC4417184; doi:10.1093/nar/gkv290)
Supplement: SUPPLEMENTARY DATA [file supp_gkv290_nar-03670-r-2014-File007.pdf]

Supplementary Material

A universal strategy for regulating mRNA translation in prokaryotic and eukaryotic cells

*Jicong Cao, Manish Arha, Chaitanya Sudrik, Abhirup Mukherjee, Xia Wu, and Ravi S. Kane**

Department of Chemical and Biological Engineering, Center for Biotechnology and Interdisciplinary Studies, Rensselaer Polytechnic Institute, Troy, NY 12180, United States.

*To whom correspondence should be addressed:
Ravi S. Kane, CBIS 4105, Rensselaer Polytechnic Institute, 110 8th Street, Troy, NY 12180, United States.
Email: kaner@rpi.edu. Tel: 518-276-2536.

Contents:

1. Supplementary Figures S1-5

2. Supplementary Tables S1-5

3. References

a

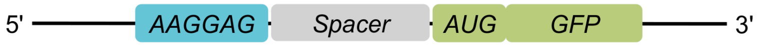

Reporter	Spacer length	Sequence
NRE(10)-GFP	10	--AAGGAGUUGUAUAUAUAUG--
NRE(11)-GFP	11	--AAGGAGAUUGUAUAUAUAUG--
NRE(12)-GFP	12	--AAGGAGAUUGUAUAUAUUAUG--

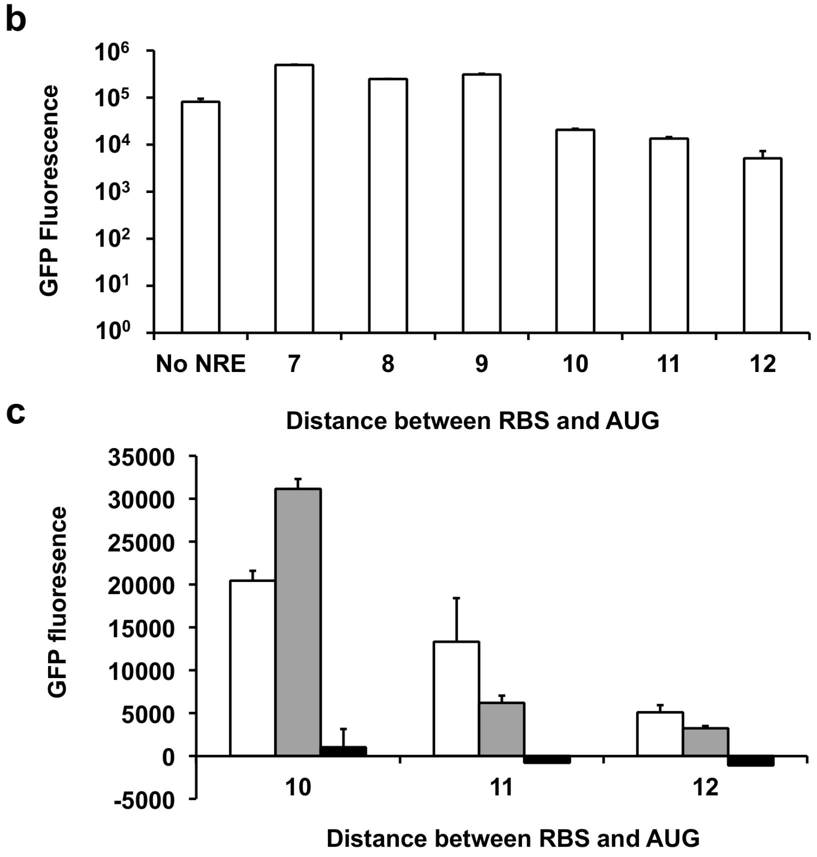

Figure S1. The influence of the spacer length on translational repression. a) The construction of the GFP reporter and the RNA sequence of the 5'-UTR of the reporters NRE(10)-GFP, NRE(11)-GFP and NRE(12)-GFP, which contain 10, 11, and 12 nucleotides, respectively, in between the RBS and AUG. The sequence of NRE is underlined. b) The normalized intensity of GFP fluorescence for cells transformed with different GFP reporters in the absence of PUF. c) The influence of PUF on the expression of GFP. The experiments were performed without regulator (white bars); with lacZ (gray bars); and with PUF (black bars). Values are the means of three independent experiments. Error bars show standard deviation.

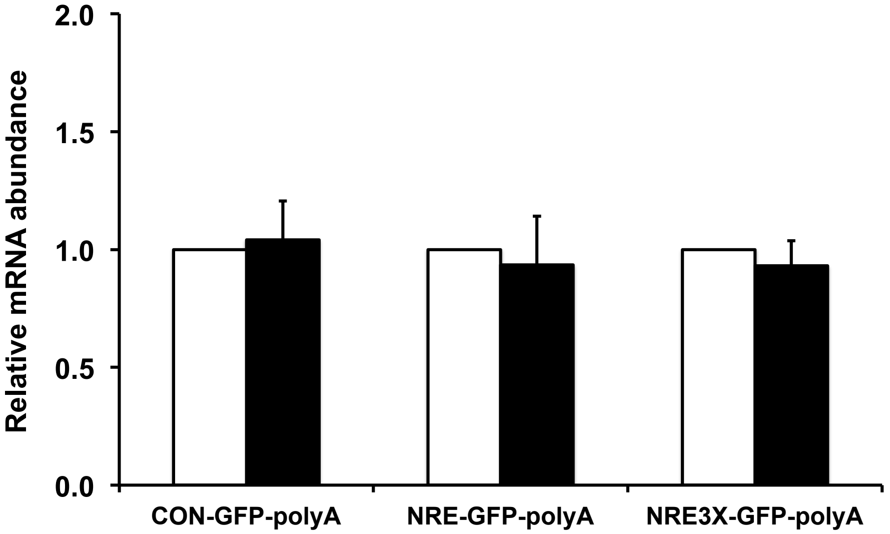

Figure S2. The influence of PUF on the transcript level of the reporter mRNA analyzed using QRT-PCR. The experiments were performed without PUF (white bars); with PUF (black bars). Relative mRNA abundance was calculated as the ratio of the normalized reporter transcript level in the absence of regulator to that in the presence of PUF. Values are the means of three independent experiments. Error bars show standard deviation.

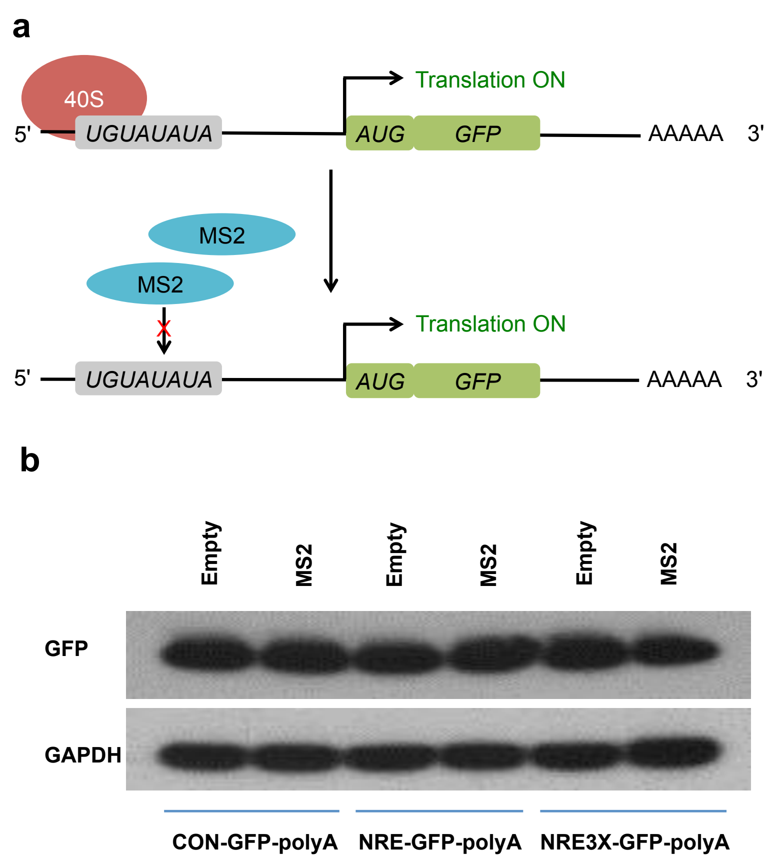

Figure S3. Influence of MS2 coat protein on translation in HEK 293T cells. a) MS2 coat protein does not bind to the PUF binding site located on the 5'-UTR of the reporter and is therefore not expected to repress the translation. b) Repression of translation analyzed using immunoblotting. Cell lysate containing 10 µg protein was loaded in each lane.

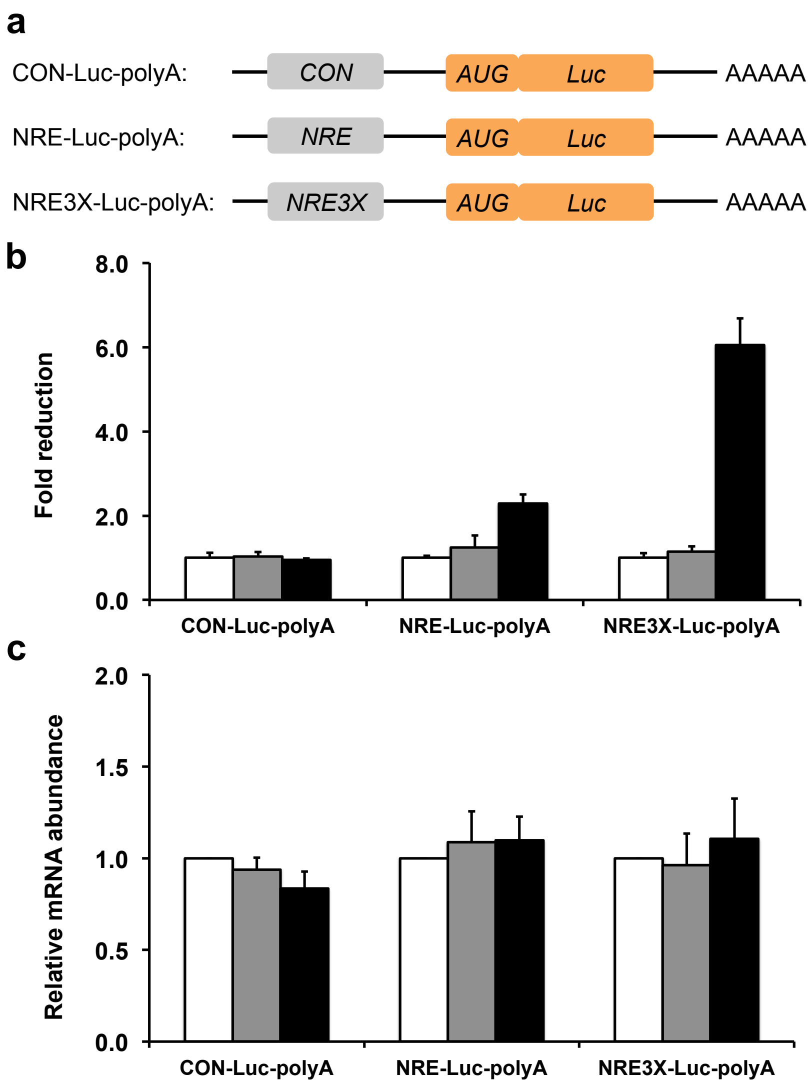

Figure S4. Repression of luciferase mRNA translation relying on the PUF-NRE interaction in HEK 293T. a) The construction of the Luc reporter and the RNA sequence of the 5'-UTR of the reporter. b) The influence of the PUF on the expression of luciferase. The reporter plasmid NRE-Luc-polyA contains one NRE, the reporter plasmid NRE3X-Luc-polyA contains three NREs, whereas the control reporter plasmid CON-Luc-polyA contains three CONs The experiments were performed without regulator (white bars); with lacZ (gray bars); and with PUF (black bars). Fold reductions were calculated as the ratio of normalized Luc fluorescence for the reporter alone to that in the presence of PUF or LacZ. c) The influence of PUF on the transcript level of the reporter mRNA analyzed using QRT-PCR. Relative mRNA abundance was calculated as the ratio of the normalized reporter transcript level in the absence of regulator to that in the presence of LacZ or PUF. Values are the means of three independent experiments. Error bars show standard deviation.

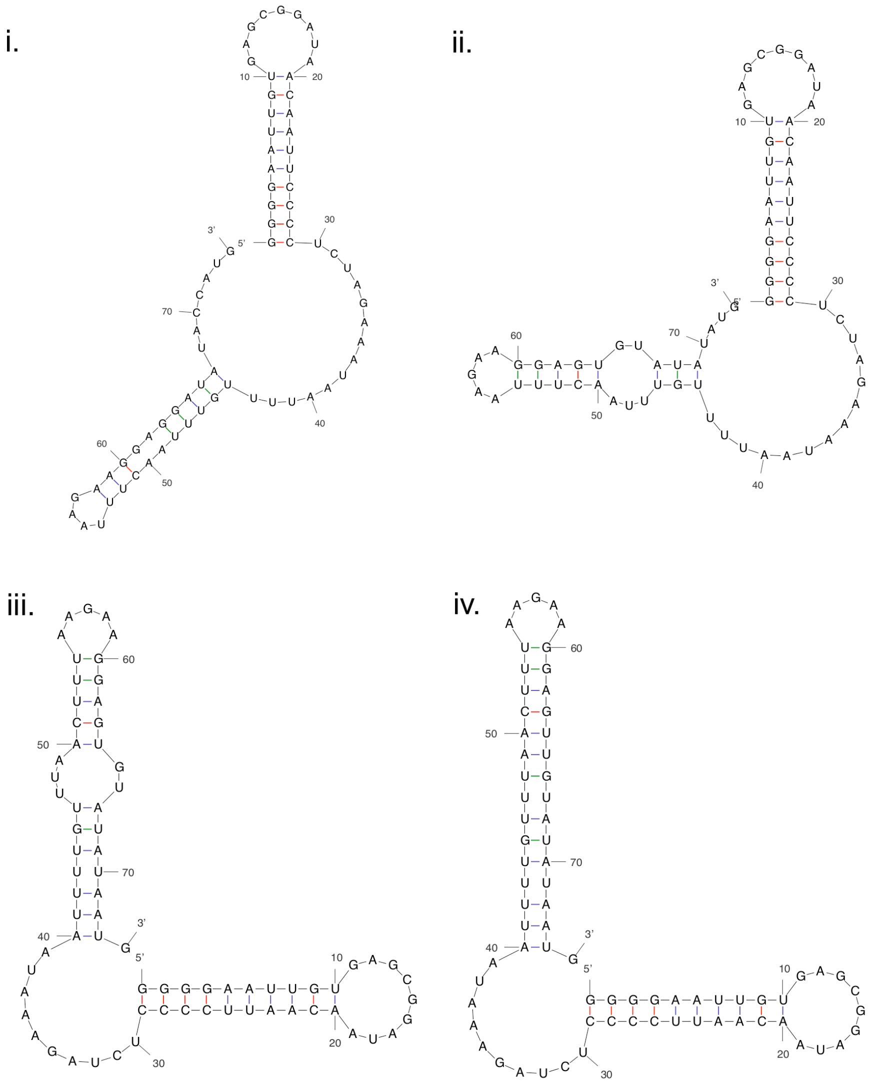

Figure S5. Secondary structures of 5'-UTR of the reporters (i) CON-GFP, (ii) NRE(7)-GFP, (iii) NRE-GFP, and (iv) NRE(9)-GFP, predicted by mfold (http://mfold.rna.albany.edu/)(1–3)

Table S1. Primers used in this study.

Primer	Template	Sequence
For PUM-pRSF Duet1	pTYB3-PUM1-HD(4)	5'CCATGGGACGCAGCCGCCTTT 3'
Rev PUM-pRSF Duet1	pTYB3-PUM1-HD	5'GGATCCTTACCCTAAGTCAACACCGTTCTTCATGTAGTACTTCTCC 3'
For CON-GFP	pEGFP-C1	5'TCTAGAAATAATTTTGTTTAACTTTAAGAAGGAGGATATACCATGGGCAGCAGCCATCACCATCATCACCACGTGAGCAAGGGCGAGGAG 3'
For NRE(7)-GFP	pEGFP-C1	5'TCTAGAAATAATTTTGTTTAACTTTAAGAAGGAGTGTATATATGGGCAGCAGCCATCACCATCATCACCACGTGAGCAAGGGCGAGGAG 3'
For NRE-GFP	pEGFP-C1	5'TCTAGAAATAATTTTGTTTAACTTTAAGAAGGAGTGTATATAATGGGCAGCAGCCATCACCATCATCACCACGTGAGCAAGGGCGAGGAG 3'
For NRE(9)-GFP	pEGFP-C1	5'TCTAGAAATAATTTTGTTTAACTTTAAGAAGGAGTGTATATATATGGGCAGCAGCCATCACCATCATCACCACGTGAGCAAGGGCGAGGAG 3'
For NRE(10)-GFP	pEGFP-C1	5'TCTAGAAATAATTTTGTTTAACTTTAAGAAGGAGTTGTATATATATGGGCAGCAGCCATCACCATCATCACCACGTGAGCAAGGGCGAGGAG 3'

For NRE(11)-GFP	pEGFP-C1	5’TCTAGAAATAATTTTGT<br>TTAACTTTAAGAAGGAG<br>TTGTATATATTATGGGCA<br>GCAGCCATCACCATCAT<br>CACCACGTGAGCAAGGG<br>CGAGGAG 3’
For NRE(12)-GFP	pEGFP-C1	5’TCTAGAAATAATTTTGT<br>TTAACTTTAAGAAGGAG<br>ATTGTATATATTATGGGC<br>AGCAGCCATCACCATCA<br>TCACCACGTGAGCAAGG<br>GCGAGGAG 3’
Rev GFP-LVA	pEGFP-C1	5’GGATCCTTAAGCTACT<br>AAAGCGTAGTTTTCGTC<br>GTTTGCTGCCTTATACA<br>GCTCGTCCATGCCGA 3’
For CON-mCherry	pmCherry-N1	5’GGATCCATTGTACACG<br>GCCGCATAATCGCATCT<br>TAGTATATTAGTTAAGTA<br>TAAGAAGGAGGATATAC<br>CATGGTGAGCAAGGGC<br>GAGGAG 3’
For NRE (8)-mCherry	pmCherry-N1	5’GGATCCATTGTACACG<br>GCCGCATAATCGCATCT<br>TAGTATATTAGTTAAGTA<br>TAAGAAGGAGTGTATAT<br>AATGGTGAGCAAGGGCG<br>AGGAG 3’
Rev mCherry-LVA	pmCherry-N1	5’CTCGAGCTAAGCTACT<br>AAAGCGTAGTTTTCGTC<br>GTTTGCTGCCTTGTACA<br>GCTCGTCCATGCCG 3’
For Luc	pGL3 Promoter	5’AGATCTATGTACCCAT<br>ACGATGTTCCAGATTAC<br>GCTGAAGACGCCAAAAA<br>CATAAAGAAAGG 3’
Rev Luc	pGL3 Promoter	5’CTCGAGTTACACGGCG<br>ATCTTTCCGC 3’

For PUM pVitro2	pTYB3-PUM1-HD	5’AGATCTGCCACCATGG GACGCAGCCGCCTTT 3’
Rev PUM pVitro2	pTYB3-PUM1-HD	5’AACGTTCCCTAAGTCA ACACCGTTCTTCATGTA GTACTTCTCC 3’

Table S2. DNA fragments used in this study.

DNA fragments	Template	Sequence
NRE 1X Position 1 A	None	5’AATTCCACGTCCCCTCGC CAGAATTGTATATATTCGG 3’
NRE 1X Position 1 B	None	5’GATCCCGAATATATACAAT TCTGGCGAGGGGACGTGG 3’
NRE 3X Position 1 A	None	5’AATTCCACGTCCCCTCGC CAGAATTGTATATATTCGCC AGAATTGTATATATTCGCCA GAATTGTATATATTCG G 3’
NRE 3X Position 1 B	None	5’GATCCCGAATATATACAAT TCTGGCGAATATATACAATT CTGGCGAATATATACAATTC TGGCGAGGGGACGTG G 3’
NRE 1X Position 9 A	None	5’AATTCCACGTCCCCTCGC AGTTCGGCCAGAATTGTAT ATATTCGG 3’
NRE 1X Position 9 B	None	5’GATCCCGAATATATACAAT TCTGGCCGAACTGCGAGGG GACGTGG 3’
NRE (6-2/7-2) 1X Position 1 A	None	5’AATTCCACGTCCCCTCGC CAGAATTTGATATATTCGG 3’
NRE (6-2/7-2) 1X Position 1 B	None	5’GATCCCGAATATATCAAAT TCTGGCGAGGGGACGTGG 3’

Table S3. Primers and TaqMan probes for QRT-PCR used in this study.

Oligonucleotides	Gene	Sequence
Sense	GFP (HEK 293T)	5'GAGGATGACGGCAACTACAA 3'
Antisense	GFP (HEK 293T)	5'CCATCTTATTGCCCAGGATGT 3'
Probe	GFP (HEK 293T)	5'-FAM490-ATACCCTGGTGAATCGCATCGAGC-BHQ-3'
Sense	Firefly luciferase (HEK 293T)	5'CGGAAAGACGATGACGGAAA 3'
Antisense	Firefly luciferase (HEK 293T)	5'CGGTACTTCGTCCACAAACA 3'
Probe	Firefly luciferase (HEK 293T)	5'-FAM490-CGTGGATTACGTCGCCAGTCAAGT-BHQ-3'
Sense	18 S (HEK 293T)	5'GTAACCCGTTGAACCCCATTC3'
Antisense	18 S (HEK 293T)	5'CCATCCAATCGGTAGTAGCGA3'
Probe	18 S (HEK 293T)	5'-CAL610-AAGTGCGGGTCATAAGCTTGCG-BHQ-3'
Sense	GFP (*E. coli*)	5’ AGGAGCGCACCATCTTCTTCA 3’
Antisense	GFP (*E. coli*)	5’ TGTCGCCCTCGAACTTCAC 3’
Probe	GFP (*E. coli*)	5’-FAM490-AGGACGACGGCAACTACAAGACCCG-BHQ-3’
Sense	16 S (*E. coli*)	5’ CGTCAGCTCGTGTTGTGAA 3’
Antisense	16 S (*E. coli*)	5’ GGACCGCTGGCAACAAAG 3’
Probe	16 S (*E. coli*)	5’-CAL610-TGTTGGGTTAAGTCCCGCAACGA-BHQ-3’

Table S4. Quantitative Analysis of Translational Repression Using Immunoblotting (Fig. 5c)

Lane No.	Reporter	Regulator	GFP	GAPDH	Fold reduction*
1	CON-GFP-polyA	Empty	5910	12360	1.0
2	CON-GFP-polyA	LacZ	7350	13029	0.8
3	CON-GFP-polyA	PUF	6901	12491	0.9
4	NRE-GFP-polyA	Empty	9771	13150	1.0
5	NRE-GFP-polyA	LacZ	9661	10185	0.8
6	NRE-GFP-polyA	PUF	1866	9359	3.7
7	NRE3X-GFP-polyA	Empty	8608	9862	1.0
8	NRE3X-GFP-polyA	LacZ	8695	10072	1.0
9	NRE3X-GFP-polyA	PUF	626	9345	13.0

* Fold reduction was calculated by dividing the ratio of the band intensity of GFP and GAPDH (I_{GFP}/I_{GAPDH}) in the absence of regulator by the ratio (I_{GFP}/I_{GAPDH}) in the presence of regulator.

Table S5. Quantitative Analysis of Translational Repression Using Immunoblotting (Fig. S3b)

Lane No.	Reporter	Regulator	GFP	GAPDH	Fold reduction*
1	CON-GFP-polyA	Empty	13430	10001	1.0
2	CON-GFP-polyA	MS2	12292	11190	1.2
3	NRE-GFP-polyA	Empty	13042	11097	1.0
4	NRE-GFP-polyA	MS2	12664	10650	1.0
5	NRE3X-GFP-polyA	Empty	14761	10751	1.0
6	NRE3X-GFP-polyA	MS2	13560	12512	1.3

* Fold reduction was calculated by dividing the ratio of the band intensity of GFP and GAPDH (I_{GFP}/I_{GAPDH}) in the absence of regulator by the ratio (I_{GFP}/I_{GAPDH}) in the presence of regulator.

REFERENCES

1. Zuker,M. (2003) Mfold web server for nucleic acid folding and hybridization prediction. *Nucleic Acids Res.* **31**, 3406-3415.
2. Waugh,A., Gendron,P., Altman,R., Brown,J.W., Case,D., Gautheret,D., Harvey,S.C., Leontis,N., Westbrook,J., Westhof,E., Zuker,M. and Major,F. (2002) RNAML: A standard syntax for exchanging RNA information. *RNA* **8**, 707-717.
3. Zuker,M. and Jacobson,A.B. (1998) Using Reliability Information to Annotate RNA Secondary Structures. *RNA* **4**, 669-679.
4. Wang,X., Zamore,P.D. and Hall,T.M. (2001) Crystal structure of a Pumilio homology domain. *Mol. Cell* **7**, 855-865.
